# Genetic Variation in the Glycoprotein B Sequence of Equid Herpesvirus 5 among Horses of Various Breeds at Polish National Studs

**DOI:** 10.3390/pathogens10030322

**Published:** 2021-03-09

**Authors:** Karol Stasiak, Magdalena Dunowska, Steven Trewick, Jerzy Rola

**Affiliations:** 1 Department of Virology, National Veterinary Research Institute, 24-100 Pulawy, Poland; karol.stasiak@piwet.pulawy.pl; 2 School of Veterinary Science, Massey University, Palmerston North 4442, New Zealand; M.dunowska@massey.ac.nz; 3 School of Agriculture and Environment, Massey University, Palmerston North 4442, New Zealand; S.Trewick@massey.ac.nz

**Keywords:** equid herpesvirus 5, genetic variability, glycoprotein B, phylogeny

## Abstract

Equid herpesvirus 5 (EHV-5) is one of two γ-herpesviruses that commonly infect horses worldwide. The objective of the study was to estimate the genetic variability within EHV-5 viruses circulating among horses in Poland. Partial glycoprotein B (gB) sequences from 92 Polish horses from 13 studs throughout Poland were compared to each other and to three EHV-5 sequences from other countries. Despite the overall high level of conservation, considerable variability was observed around the putative furin cleavage site. Based on phylogenetic analysis, the viruses clustered within two major lineages (A and B), with further sub-clustering within group A. The clustering of EHV-5 sequences was independent of age or geographical origin of the sampled horses. Recombination was identified as one of the factors contributing to the genomic heterogeneity. Viruses from unweaned foals were more similar to viruses from other foals at the same stud than to viruses form their dams, suggesting the horizontal transfer and/or evolution of EHV-5 within individual hosts. Our data indicate that the gB sequence is not suitable for tracking the source of EHV-5 infection. Further research is needed to elucidate the importance of the sequence variability around the EHV-5 gB furin cleavage site on the biology of the virus.

## 1. Introduction

Horses are natural hosts to five herpesviruses, including equid herpesvirus (EHV) type 5 [1]. This virus is classified in the family *Herpesviridae*, subfamily *Gammaherpesvirinae*, and genus *Percavirus* [2]. It shares sequence similarity with another equine gammaherpesvirus EHV-2, as well as with human herpesvirus 4 (Epstein–Barr virus, EBV) [3]. It has been detected from horses worldwide with the higher frequency of detection in young horses compared to older ones [4,5,6]. Following primary infection, which often occurs early in life, the virus establishes lifelong latent infection in B lymphocytes [7].

The pathogenic potential of EHV-5 has not been fully elucidated, as the virus has been detected from healthy horses as well as from horses with varied clinical presentations including poor performance syndrome, mild respiratory disease, pneumonia, and ocular disease [1,8,9]. The most serious, albeit rare, disease that has been linked to EHV-5 infection in mature horses is equine multinodular pulmonary fibrosis [10,11]. 

Very limited genomic data are currently available for EHV-5, with only one fully sequenced genome of the Australian prototype isolate EHV-5 2-141/67 deposited in GenBank [12]. The linear, double-stranded DNA genome of EHV-5 is 179 kbp long and consists of the 79 functional open reading frames (ORFs) with a mean G + C content of 52% [12,13]. Typically for herpesviruses, family-conserved genes are interspersed with species-specific genes and non-coding regions [3,12]. The conserved herpesviral genes include a gene coding for glycoprotein B (gB). Glycoprotein B is a highly conserved glycoprotein with homologues among all members of the family *Herpesviridae* [14]. It plays a pivotal role in the herpesvirus life cycle, including virus entry and cell-to-cell spread [15]. The gB of both EHV-2 and EHV-5 is a disulfate-linked heterodimer that forms an integral part of the viral envelope [16,17]. Glycoprotein B has been commonly used in phylogenetic analysis of herpesviral genomes [18]. 

Previously, we have reported the high prevalence of equid γ-herpesvirus infections among Polish horses of various breeds and ages [19]. As part of that study, we also reported considerable heterogeneity of Polish EHV-2 sequences, which was in agreement with similar data from other countries [20,21]. Limited data are available for EHV-5 genomic variability, but results from some studies suggest that the genomic heterogeneity of EHV-5 is lower than that observed for EHV-2 [22,23]. However, others showed high genomic heterogeneity of the analyzed EHV-5 sequences [24,25,26,27]. Only two studies investigated the changes in EHV-5 genotypes obtained from individual horses over time [27,28]. Both showed that genotypes varied between individual horses and that more than one genotype could be occasionally recovered from the same nasal swab sample. The most recent and the largest of the studies investigating EHV-5 genomic heterogeneity involved phylogenetic analysis of approximately 460 bp of gB from 34 EHV-5 sequences [28]. Based on this analysis, the authors proposed the existence of four main genotype groups labeled I to IV, with <2% variability at the nucleotide level within each group. 

The objective of the current study was to estimate the genetic variability within EHV-5 circulating among horses in Poland and to compare these with EHV-5 sequences from other countries.

## 2. Results

### 2.1. Sequence Analysis

Out of 100 EHV-5 positive samples included in the study, eight were removed from the analysis due to insufficient quality of the amplified gB sequence. Partial (1.3 kbp) gB gene sequences from the remaining 92 Polish EHV-5 had between 89.9% and 100% identity at the nucleotide level and between 88.1% and 100% at the amino acid level. Overall, 59/92 (64.1%) predicted amino acid sequences were unique. The degree of identity between Polish EHV-5 sequences and three international sequences was similar and ranged from 89.5% to 99.8% at the nucleotide level and from 87.5% to 100% at the amino acid level.

The 5′ (nn 404–1126 in AF050671) and 3′ (nn 1433–1621 in AF050671) ends of the analyzed sequences were comparatively conserved, with an average pairwise percentage identity of 99.6% and 99.7% for the 5′ and 3′ ends, respectively. The corresponding number of identical sites was 224/241 (92.9%) and 60/63 (95.2%), respectively. In contrast, the region around the putative furin cleavage site (nn 1127 to 1432 in AF050671) was highly variable, with only 51/110 (46.4%) identical sites and 77.1% average mean pairwise identity.

**Figure 1:**
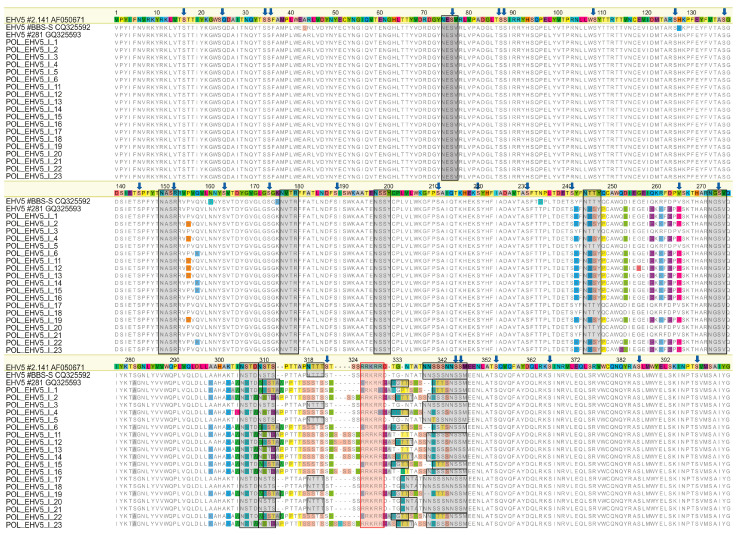
Alignment of deduced partial glycoprotein B sequence from equid herpesvirus 5 (EHV-5) from Arabian horses in stud I including three mare/foal pairs (horses 1/2, 3/4, and 5/6). Two EHV-5 sequences from Iceland and one from Australia are included for comparison. Residues that differ from those in the reference Australian sequence (strain 2-141/67) are highlighted. The putative furin cleavage site is marked with a red box. Gray boxes indicate putative N-glycosylation sites and arrows point to conserved cysteine residues.

The putative furin cleavage site (R/K-R-K-R-R/K, aa 422–426 in AIU39535.1) was conserved among all sequences and consistent with a canonical furin cleavage motif (R-X-K/R-R↓) for all but one sequence (POL_EHV-5_I_1), where KRKRK was present. Between 10 and 14 N-glycosylation sites were identified in individual predicted gB sequences (Figure 1). 

The position of seven N-terminal sites was highly conserved between all analyzed sequences, although in two instances (aa 244–248 and 305–309 in alignment in Figure 1), the aa sequence associated with the predicted glycosylation sites varied between viruses. 

**Figure 2:**
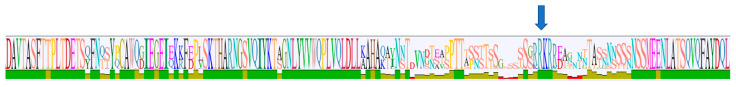
Amino acid sequence logo around the putative furin cleavage site, as indicated by a blue arrow (aa 422–426 in AF050671). The region included in the logo spans aa 226 to 358 in AF050671. The logo was generated from the alignment of 95 partial EHV-5 glycoprotein B sequences including 92 sequences from the current study and three international sequences (accession numbers AF050671, GQ325592, and GQ325593). The graph underneath the logo shows the mean pairwise identity of all pairs in the columns (green: 100%, brown: more than 30% but less than 100%, red: less than 30%).

One additional putative N-glycosylation site (NSSM, aa 344–348 in Figure 1) was conserved between all sequences. The position of predicted N-glycosylation sites around the cleavage sites was more variable, with three to six sites identified in that region in different sequences (Figure 1). There were 26 cysteine residues conserved between all 95 sequences. Amino acid alignment of a subset of sequences comprising sequences from Stud I (including three mare/foal pairs) and three EHV-5 sequences from GenBank is shown in Figure 1, and the sequence logo of the variable region around the furin cleavage site generated from the full alignment of 95 sequences in shown in Figure 2.

### 2.2. Phylogeny

Based on the phylogenetic tree constructed from alignment of the full available sequence (≈1.2 kbp) of EHV-5 gB (Figure S1), the 95 EHV-5 sequences clustered within two main lineages. Most (*n* = 58) were clustered within a large group designated group A that could be further divided into at least three sub-clusters containing 42 (cluster A1), 3 (cluster A2), and 13 (cluster A3) EHV-5 sequences. 

Cluster A3 included EHV-5 isolate 281 from Iceland. The second group (B) contained 37 EHV-5 sequences including two overseas EHV-5 sequences (Australian 2-141/67 and Icelandic BB5-5). Based on the phylogenetic tree inferred from the alignment of a shortened fragment of EHV-5 that included an additional 27 EHV-5 Swedish and three British EHV-5 sequences previously analyzed by Back and others [28], sequences from groups designated by Back as I and II clustered with sequences from group A1 in the current analysis, while sequences from Back’s groups III and IV clustered with sequences from groups A2 and B, respectively (Figure S1).

**Figure 3:**
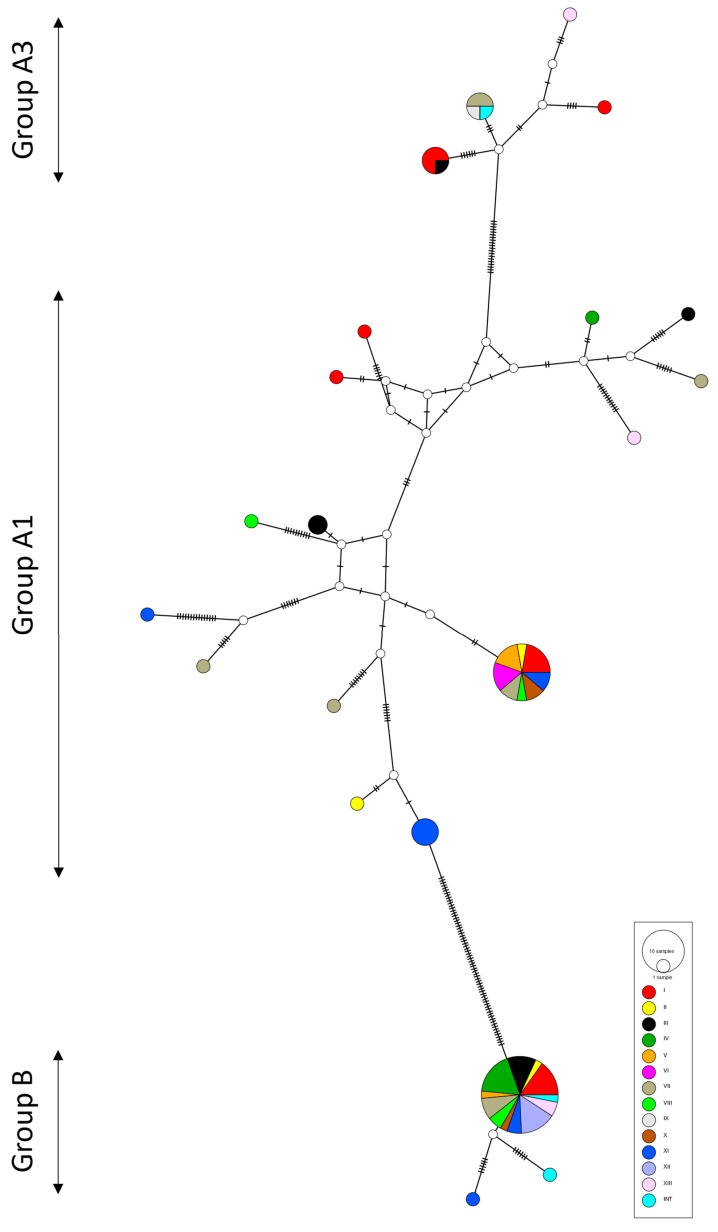
Population genetic structure of equid herpesvirus type 5 (EHV-5) sequences (*n* = 66) including sequences (*n* = 78) from Polish horse studs (I through to XIII) obtained in the current study and sequences from GenBank (*n* = 3) from international (INT) locations (Australia and Iceland). The number of nucleotide substitutions between haplotypes is represented by ticks on branches. Nodes are scaled based on the number of representative sequences and colored based on the geographic location of EHV-5 positive horses. Small open white circles indicate inferred nodes that are not represented among sequences included in the network. Reticulation in a haplotype network indicates that the data did not contain an appropriate signal to resolve a single pathway through a network.

Three putative recombination events detected in 14 sequences were supported by four (two events) or five (one event) out of five default methods used within the RDP software. However, the exact position of breakpoints was not identified for any of those events. The non-recombinant Polish EHV-5 sequences were between 89.9% and 100% identical to each other at the nucleotide level and between 88.1% and 100% identical at the amino acid level. They clustered within three groups (Figure 3) corresponding to groups A1, A3, and B in the ML (Figure 3). 

Sequences from cluster A2 were identified as putative recombinants and hence were not included in the network analyses. The haplotype network structure did not correlate with horse age group or with the geographical location of the horses sampled, which was supported by the results of AMOVA (Table 1).

## 3. Discussion

We have presented the largest and the most comprehensive study of variability between EHV-5 gB sequences. Our dataset included 92 viruses obtained from horses of different ages and breeds from throughout Poland and three international sequences available in GenBank. Overall, the clustering of EHV-5 in the current study was similar to that reported by Back and others [28] based on the analysis of a shorter (≈450 bp) gB fragment from a smaller number of horses from one training yard, with the separation of EHV-5 sequences into at least three groups: A1, A3, and B, corresponding to groups I/II, III, and IV, respectively, in the Back’s paper [28]. Group A1 was the most heterogeneous of the three, which was represented by the extensive reticulation present in the population network (Figure 3).

The clustering did not appear to correlate with the geographical origin of the virus or the age of horses, as samples from different studs and horses from different age groups were distributed throughout the phylogenetic tree or haplotype network. As such, the gB sequence is unlikely to be useful for epidemiological studies that aim to determine the source of infection or the pathways of virus spread between horses. This can be well illustrated by the fact that two Icelandic EHV-5 sequences clustered within two different groups, each interspersed with Polish EHV-5 sequences. This is despite the fact that Icelandic horses have been isolated from the rest of the word’s equine population for centuries [29].

Glycoprotein B was chosen for the analysis, as it is highly conserved among all herpesviral genomes [14]. Despite the overall high level of conservation, considerable variability was observed within the central region of the analyzed EHV-5 gB sequences (aa 242–343 in Figure 1). This is consistent with data reported by others, who described that gB sequences from rhadinoherpesviruses were least conserved within the N-terminal end (first 49 aa in EHV-5 strain 2-141), the C-terminal end (from aa 769 in EHV-5 strain 2-141), and in the central region around the furin cleavage site [16,30].

Although the sampling strategy employed in the current study did not allow for a comparison of samples from diseased and healthy horses from the same stud, EHV-5 sequences from diseased horses from studs II and III did not cluster together. This is in agreement with the lack of correlation between the EHV-5 gB sequence and health status that has been recently reported among a group of two- to five-year-old Swedish race horses from one training yard based on the analysis of a shorter fragment of EHV-5 gB [28]. The relationship between gB genotypes and clinical outcomes deserves further focus in future investigations, as it is possible that this relationship is more complex than the design of the current study allowed to explore. For example, Coaquette and others [31] described that infection with multiple gB genotypes of human cytomegalovirus (HCMV), rather than the presence of a single specific gB genotype, was a critical factor associated with severe clinical manifestations of HCMV-associated disease in immunocompromised patients.

It was clear from the phylogenetic analysis that several different EHV-5 genotypes circulated among horses at several studs. Sequences from some studs (e.g., Stud I or III) were distributed across the entire tree, within all four main lineages. Sequences from other studs appeared less variable. For example, all five sequences from Stud XII and 6/7 sequences from Stud IV clustered within group B (Figure S1). As horses from different studs belonged to different breeds, this may suggest the existence of some breed-related differences. However, it is equally possible that such differences represent differences in the number of viral genotypes circulating among horses or the number and management of horses sampled at each stud.

Even within studs with highly variable EHV-5 sequences, some horses were infected with viruses that were identical to each other over the length of the analyzed gB fragment suggesting a horizontal spread. This can be exemplified by EHV-5 sequences from horses 17, 18, and 20 at Stud I, sequences 83, 86, 87, and 88 at Stud XI, or sequences 89 and 92 at Stud XII. However, sequences from horses 26 (Stud II) and 38 (Stud IV) were also 100% identical to each other, even though they originated from horses from different studs and breeds that were unlikely to have any links with each other.

In contrast, somewhat surprisingly, viruses obtained from foals 2, 4, and 6 at Stud I showed more similarity to EHV-5 obtained from other foals at that stud than to viruses from their dams, despite the fact that the foals were co-housed with their dams during the night. For example, EHV-5 from foal 6 was only 90.4% identical to EHV-5 from its dam (horse 5), but it was 99.9% identical to EHV-5 sequences from foals 15 and 21 (Figure 1). The foals and mares were turned out together in a pasture for some time during the day, which may have provided an opportunity for the foals to acquire EHV-5 infection from other horses.

It is also possible that foals may have been infected with several different EHV-5 viruses including those from their dams, but the predominant virus that was detected represented a genotype that was different from the predominant genotype maintained by the dam. The hallmark of herpesvirus infection is the establishment of latency following the primary infection. Latently infected horses are considered to be infected for life and can periodically re-activate the virus with or without accompanying clinical signs of disease [9]. The immunological correlates that are linked to the establishment of and recrudescence from latency are currently poorly understood, but the interactions between herpesviruses and the immune system of their hosts are complex. Therefore, it is possible that such interactions drive the selection of the most successful EHV-5 genotype within an individual horse. The relative stability of EHV-5 genotypes obtained from individual horses over a period of weeks to months has been described previously [27,28]. The same authors also occasionally detected multiple variants of EHV-5 from the same horse. The comparatively high variability of the EHV-5 sequences analyzed in the current study, combined with the lack of clustering according to the geographical location, supports the possibility of selection of unique EHV-5 variants within individual horses. It is unlikely that such variability could be explained by the movement of horses or fomites between studs, particularly between those with different breeds. If so, factors such as genetic background, the diversity of viruses circulating in the area, or the immune status of the horse are likely to be important in the interactions between EHV-5 and each infected horse.

The results of RDP analysis suggested that at least some of the variability observed between EHV-5 sequences in the current study was due to recombination. These results should be interpreted with caution, as none of the recombination events was supported by all five methods used within default settings in RDP, with no clear identification of the breakpoints. This was most likely due to the fact that none of the recombination detection methods can reliably detect recombination in poorly aligned regions. For this reason, all poorly aligned regions were removed from the alignment for RDP analysis, which included the highly variable sites around the putative furin cleavage site. Detection of recombination is also difficult, or even impossible, between highly similar sequences. Despite these limitations, recombination was identified in 12 sequences. This was not surprising as recombination has been shown to contribute to the genomic variability observed among other herpesviruses [32,33,34]. However, the removal of putative recombinant sequences from the analyses did not reduce the level of variability observed between the remaining EHV-5 sequences, suggesting that mechanisms other than recombination must have contributed to the evolution of those sequences.

It has been shown previously that EHV-5 gB is incorporated into the viral envelope in the cleaved form, with the sizes of the cleaved fragments consistent with furin cleavage [16]. All 92 predicted gB sequences from Polish EHV-5 had a conserved putative furin cleavage site, which was consistent with reports by others using smaller datasets [16,28]. Although the canonical motif for furin cleavage has been described as R-X-[K/R]-R↓, several variations to this motif have been recognized, including R-X-R-K, which is present in at least one of the Polish sequences [35]. Furin cleavage plays a role in a variety of biological processes including the facilitation of infection by viruses from several families including *Herpesviridae* [36], *Coronaviridae* [37], *Papillomaviridae* [38], or *Pneumoviridae* [39]. The region around the furin cleavage site has been shown to be under positive selection during infection with human cytomegalovirus [40] despite the fact that proteolytic processing by furin is not essential for HCMV growth in cell culture [41]. The conservation of this site among all 95 EHV-5 sequences included in the analysis suggest that furin cleavage is important for EHV-5 gB processing during EHV-5 infection, but the exact effects of furin cleavage on EHV-5 life cycle remain to be determined.

Ten cysteine residues that form five disulfide linkages bonds important for the three-dimensional structure of herpesviral gB are conserved among gB homologs, five of which are located within the fragment analyzed in the current study [14,16,42]. All five were conserved between all 95 EHV-5 sequences analyzed (aa positions 52, 117, 201, 249 and 379 in Figure 1). Holloway and others [16] also reported that EHV-5 gB was N-glycosylated and contained 16 putative N-glycosylation sites, three of which were conserved between sequences of nine γ-herpesviruses analyzed in that study (aa 170, 554, and 630 in AF050671). One of those predicted N-glycosylation sites (aa 170, corresponding to position 74 in Figure 1) was located within the fragment analyzed in the current study and was conserved between all 95 sequences. Several other putative N-glycosylation sites were conserved between all EHV-5 sequences in the current study, with some variability between the exact position of N-glycosylation sites around the putative furin cleavage site, highlighting the importance of N-glycosylation for the processing and function of herpesviral gB [16,17,43].

The methodology employed in the current study did not allow for detection of multiple genotypes from the same sample, as PCR products were sequenced directly, without prior cloning or using next-generation sequencing. The presence of multiple genotypes in the sample may be one possible explanation for the number of unresolved sites in sequences from eight horses that were excluded from the analysis. Another limitation of the sampling strategy is a lack of multiple samples from individual horses over time.

In summary, we have presented a comprehensive analysis of a large set of partial EHV-5 gB sequences. Our data further supported the presence of conserved features in EHV-5 gB sequences. However, there was also a considerable variation between EHV-5 sequences from individual horses, even among horses that have been in close contact with each other. The sequence variability was concentrated around the putative furin cleavage site, and it was not correlated with geographical origin or the age of sampled horses. The implication of the changes within this region on interactions between the virus and its host need further elucidation.

## 4. Materials and Methods

### 4.1. Sample Collection

**Figure 4:**
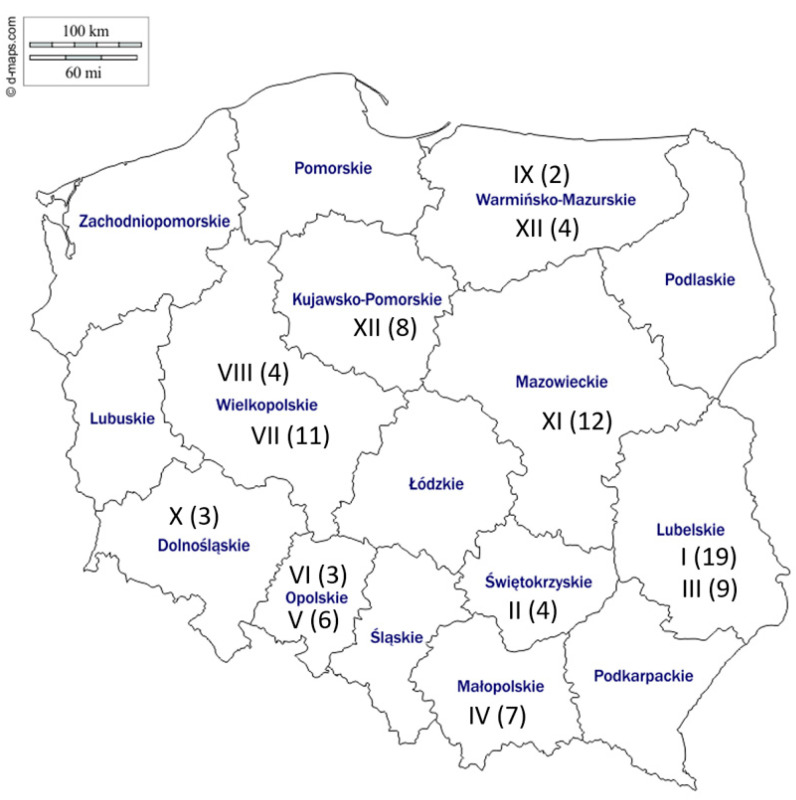
Map of Poland showing the location of the studs (denoted by Arabic numbers) and the number of EHV-5 positive horses from each stud that were included in the current study.

DNA collected from nasal swab samples obtained as part of a separate study [19] were used as a starting material. Briefly, horses (*n* = 540) of nine various breeds from 13 different studs throughout Poland (Figure 4) were sampled once between April 2015 and May 2016. 

At each stud, horses of different ages including foals, mares, yearlings and 2-year-olds were included. Horses from Studs II and III showed clinical signs of respiratory disease at the time of sampling. The remaining horses were healthy at the time of sample collection. The collection and processing of samples have been described in detail elsewhere [19]. The swabs were tested for the presence of EHV-1, EHV-4, EHV-2, and EHV-5. Nearly half (254/540, 47.0%) of the sampled horses tested positive for EHV-5, either alone or in combination with other EHVs [19]. The EHV-5 positive swabs in the current study originated from horses of different ages including foals (<1 year old, 24 samples), yearlings (33 samples), young horses (2–5 years old, 17 samples), mature horses 6–10 years old (8 samples), 11–15 years old (9 samples), and more than 15 years old (1 sample). The EHV-5 positive samples from Stud I included three foal–mare pairs (Table S1).

### 4.2. Conventional PCR and Sequencing

Viral DNA from randomly selected (*n* = 100) EHV-5 positive swabs with Cq value below 32 was used as a template in conventional PCR assay targeting a 1339 bp fragment of the gB gene [5]. Each PCR reaction was performed in a total volume of 25 µL and consisted of 1 x buffer (Sigma-Aldrich, St. Louis, LA, USA), 600 nM of each primer (forward: 5′-CCAACACAGAAGACAAGGAG-3′; reverse: 5′-CACGGTGATACAGTCAGAGA-3′), 0.2 mM deoxynucleotide mix (Sigma-Aldrich), 0.5 µL JumpStart AccuTaq LA DNA Polymerase (Sigma-Aldrich), and 2 µL of template DNA. Amplification was performed in a Biometra Thermocycler (Biometra, Göttingen, Germany) using the following cycling conditions: initial denaturation at 94 °C for 5 min, followed by 35 cycles of denaturation at 94 °C for 1 min, primer annealing at 59.5 °C for 1 min, and elongation at 72 °C for 1 min. The final elongation was extended to 7 min at 72 °C. All common precautions to avoid PCR cross-contamination (separate areas for preparation of master mix, DNA extractions and gel electrophoresis, use of filtered tips, etc.) were employed. In addition, negative non-template controls were included with each PCR run. Amplified products were subjected to electrophoresis through a 1.5% ethidium bromide stained agarose gel, purified (ExoSAP-IT PCR Product Cleanup Reagent, Thermo Fisher Scientific, Santa Clara, CA, USA), and sequenced in both directions with the same primers used for amplification using BigDye^®^ Terminator version 3.1 (Applied Biosystems, Austin, TX, USA) on a 3730 xl DNA Analyzer (Applied Biosystems) at the Genomed (Warsaw, Poland).

### 4.3. Sequence Analyses

The obtained sequences were assembled using Geneious 9.1.8 (https://www.geneious.com, accessed on 8 March 2021) and trimmed to exclude low-quality ends. The 5′ and 3′ ends of all trimmed sequences corresponded to nucleotides 404 and 1621, respectively in the sequence of the reference Australian strain EHV-5. 2-141/67 (GenBank accession AF050671). The nucleotide sequences were translation-aligned using MUSCLE amino acid alignment within Geneious v9.1.8 (Biomatters, Ltd., Auckland, New Zealand) [44]. Both full alignments of 95 sequences and an alignment of subset sequences from Stud I were generated. A phylogenetic tree was inferred using the maximum likelihood method with 500 bootstrap replicates using the K2+G+I substitution model in MEGAX (version 10.05) software [45]. An additional tree was constructed using an alignment of a shorter fragment of gB (≈450 nucleotides) in order to include Swedish (*n* = 27) and British (*n* = 3) EHV-5 sequences available in GenBank that have been used for similar analyses previously [28]. The best substitution model was identified using a model selection tool in MEGAX. The putative N-glycosylation sites were determined using ScanProsite (https://prosite.expasy.org, accessed on 8 March 2021). The amino acid sequence logo around the putative furin cleavage site was generated in Geneious. Recombination detection was performed using Recombination Detection Program (RDP) version 4.101 [46]. Poorly aligned regions with gaps had been removed from the alignment before uploading it into the RDP software. All recombination events that were supported by two or more methods were accepted. Then, putative recombinant EHV-5 sequences were removed from the alignment, and the remaining non-recombinant sequences were realigned in Geneious, exported to MEGAX, and saved as a nexus file, which was then used as an input file to generate a haplotype list in DnaSP software (available from http://www.ub.edu/dnasp/, accessed on 8 March 2021). Sites with gaps or missing/ambiguous data were excluded. Then, the sequences representative of the haplotypes were aligned in Geneious and exported in nexus format with a trait block added to represent the locations (studs) and age groups or horses. The median joining haplotype networks were drawn using default parameters in PopART version 1.6 [47]. Analysis of molecular variance (AMOVA) was used to test for correlation between the population genetic structure of the EHV-5 sequences, the geographic origin of samples (stud), and the age of horses. The strength of correlation was represented by a PhiPT value, with 0 indicating no correlation and 1 indicating perfect correlation. The corresponding P values were generated by reference to 1000 random permutations of the input data.

### 4.4. GenBank Accession Numbers

The nucleotide sequences of Polish EHV-5 described in this study were submitted to GenBank under the following accession numbers: MW526273—MW526364.

## Figures and Tables

**Table 1:** Analysis of molecular variance (AMOVA) results for population genetic structure within Polish equid herpesvirus type 5 sequences (*n* = 81). The network included international (INT) sequences from Iceland (*n* = 2) and Australia (*n* = 1) from horses of unknown (UNK) ages.

Test	Variation within Populations	Variation between Populations	PhiPT	P
Geographical location (studs I–XIII, INT)	93%	7%	0.07	0.15
Age (<1, 1, 2–5, 6–10, 11–15, >15 years, UNK)	93%	7%	0.06	0.08

## Data Availability

The data sets supporting the results of this article are included within the article. The nucleotide sequences of Polish EHV-5 described in this study were submitted to GenBank under the accession numbers: MW526273—MW526364.

## Supplementary Materials

The following are available online at https://www.mdpi.com/2076-0817/10/3/322/s1, Figure S1: The evolutionary history was inferred by using the Maximum Likelihood method and Kimura 2-parameter (K2 + G + I) model, Table S1: Source of EHV-5 viruses included in the study.
