# Supplementary material for: Genetic Variation in the Glycoprotein B Sequence of Equid Herpesvirus 5 among Horses of Various Breeds at Polish National Studs
Source: Pathogens. 2021 Mar 9;10(3):322. doi: 10.3390/pathogens10030322 (PMC7998979; doi:10.3390/pathogens10030322)
Supplement: Supplementary file 1 [file pathogens-10-00322-s001.zip › Supplementary Figure S1.pdf]

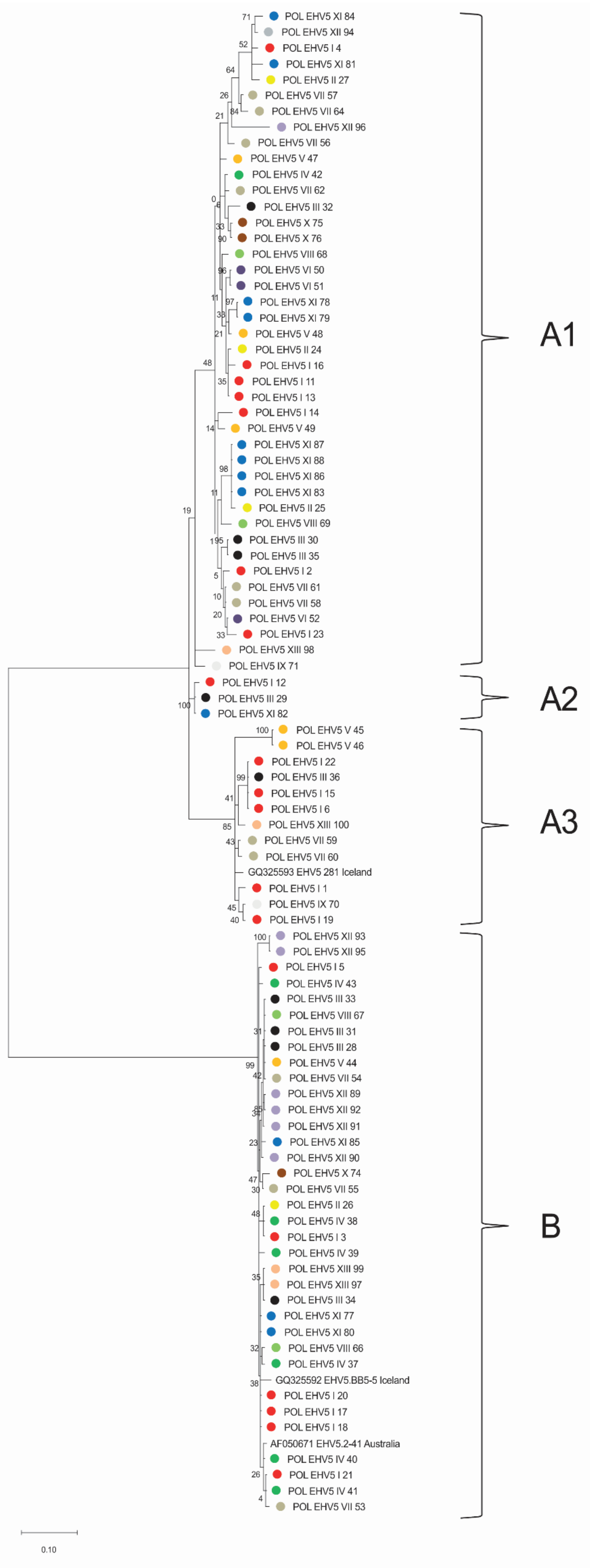
POL EHV5 XI 84
POL EHV5 XII 94
POL EHV5 I 4
POL EHV5 XI 81
POL EHV5 II 27
POL EHV5 VII 57
POL EHV5 VII 64
POL EHV5 XII 96
POL EHV5 VII 56
POL EHV5 V 47
POL EHV5 IV 42
POL EHV5 VII 62
POL EHV5 III 32
POL EHV5 X 75
POL EHV5 X 76
POL EHV5 VIII 68
POL EHV5 VI 50
POL EHV5 VI 51
POL EHV5 XI 78
POL EHV5 XI 79
POL EHV5 V 48
POL EHV5 II 24
POL EHV5 I 16
POL EHV5 I 11
POL EHV5 I 13
POL EHV5 I 14
POL EHV5 V 49
POL EHV5 XI 87
POL EHV5 XI 88
POL EHV5 XI 86
POL EHV5 XI 83
POL EHV5 II 25
POL EHV5 VIII 69
POL EHV5 III 30
POL EHV5 III 35
POL EHV5 I 2
POL EHV5 VII 61
POL EHV5 VII 58
POL EHV5 VI 52
POL EHV5 I 23
POL EHV5 XIII 98
POL EHV5 IX 71
POL EHV5 I 12
POL EHV5 III 29
POL EHV5 XI 82
POL EHV5 V 45
POL EHV5 V 46
POL EHV5 I 22
POL EHV5 III 36
POL EHV5 I 15
POL EHV5 I 6
POL EHV5 XIII 100
POL EHV5 VII 59
POL EHV5 VII 60
GQ325593 EHV5 281 Iceland
POL EHV5 I 1
POL EHV5 IX 70
POL EHV5 I 19
POL EHV5 XII 93
POL EHV5 XII 95
POL EHV5 I 5
POL EHV5 IV 43
POL EHV5 III 33
POL EHV5 VIII 67
POL EHV5 III 31
POL EHV5 III 28
POL EHV5 V 44
POL EHV5 VII 54
POL EHV5 XII 89
POL EHV5 XII 92
POL EHV5 XII 91
POL EHV5 XI 85
POL EHV5 XII 90
POL EHV5 X 74
POL EHV5 VII 55
POL EHV5 II 26
POL EHV5 IV 38
POL EHV5 I 3
POL EHV5 IV 39
POL EHV5 XIII 99
POL EHV5 XIII 97
POL EHV5 III 34
POL EHV5 XI 77
POL EHV5 XI 80
POL EHV5 VIII 66
POL EHV5 IV 37
GQ325592 EHV5.BB5-5 Iceland
POL EHV5 I 20
POL EHV5 I 17
POL EHV5 I 18
AF050671 EHV5.2-41 Australia
POL EHV5 IV 40
POL EHV5 I 21
POL EHV5 IV 41
POL EHV5 VII 53
A1
A2
A3
B
0.10

Figure S1. The evolutionary history was inferred by using the Maximum Likelihood method and Kimura 2-parameter (K2+G+I) model. The tree with the highest log likelihood (-15530.64) is shown. The percentage of trees in which the associated taxa clustered together is shown next to the branches. Initial tree(s) for the heuristic search were obtained automatically by applying Neighbor-Join and BioNJ algorithms to a matrix of pairwise distances estimated using the Maximum Composite Likelihood (MCL) approach, and then selecting the topology with superior log likelihood value. A discrete Gamma distribution was used to model evolutionary rate differences among sites (5 categories (+G, parameter = 0.1338)). The rate variation model allowed for some sites to be evolutionarily invariable ([+I], 43.59% sites). The tree is drawn to scale, with branch lengths measured in the number of substitutions per site. This analysis involved 95 nucleotide sequences. All positions with less than 90% site coverage were eliminated, i.e., fewer than 10% alignment gaps, missing data, and ambiguous bases were allowed at any position (partial deletion option). There were a total of 1209 positions in the final dataset. Evolutionary analyses were conducted in MEGA X [30]. The Polish EHV-5 sequences obtained in the current study are labelled POL_EHV5_stud number_horse ID number. Samples from the same stud are labelled with the rectangle of the same color. Accession numbers for sequences from GenBank are included in the description of each sequence.
