# Supplementary material for: Genetic Variation in the Glycoprotein B Sequence of Equid Herpesvirus 5 among Horses of Various Breeds at Polish National Studs
Source: Pathogens. 2021 Mar 9;10(3):322. doi: 10.3390/pathogens10030322 (PMC7998979; doi:10.3390/pathogens10030322)
Supplement: Supplementary file 1 [file pathogens-10-00322-s001.zip › Supplementary Table S1.pdf]

Table S1. Source of EHV-5 viruses included in the study.

No	Sequence ID	Horse breed[1]	Stud farm	Age Group (years)	Respiratory disease	Putative recombinant
1	POL_EHV5_I_1	Arabian (M1)	I	11 - 15	No	No
2	POL_EHV5_I_2	Arabian (F1)	I	< 1	No	No
3	POL_EHV5_I_3	Arabian (M2)	I	6 - 10	No	No
4	POL_EHV5_I_4	Arabian (F2)	I	< 1	No	Yes
5	POL_EHV5_I_5	Arabian (M3)	I	2 - 5	No	No
6	POL_EHV5_I_6	Arabian (F3)	I	< 1	No	No
7	POL_EHV5_I_11	Arabian	I	< 1	No	No
8	POL_EHV5_I_12	Arabian	I	< 1	No	Yes
9	POL_EHV5_I_13	Arabian	I	< 1	No	No
10	POL_EHV5_I_14	Arabian	I	< 1	No	No
11	POL_EHV5_I_15	Arabian	I	< 1	No	No
12	POL_EHV5_I_16	Arabian	I	< 1	No	No
13	POL_EHV5_I_17	Arabian	I	< 1	No	No
14	POL_EHV5_I_18	Arabian	I	< 1	No	No
15	POL_EHV5_I_19	Arabian	I	< 1	No	No
16	POL_EHV5_I_20	Arabian	I	< 1	No	No
17	POL_EHV5_I_21	Arabian	I	1	No	Yes
18	POL_EHV5_I_22	Arabian	I	1	No	No
19	POL_EHV5_I_23	Arabian	I	1	No	No
20	POL_EHV5_II_24	Arabian	II	< 1	Yes	No
21	POL_EHV5_II_25	Arabian	II	< 1	Yes	No
22	POL_EHV5_II_26	Arabian	II	< 1	Yes	No
23	POL_EHV5_II_27	Arabian	II	6 - 10	Yes	Yes
24	POL_EHV5_III_28	Arabian	III	1	Yes	No
25	POL_EHV5_III_29	Arabian	III	1	Yes	Yes
26	POL_EHV5_III_30	Arabian	III	1	Yes	No
27	POL_EHV5_III_31	Arabian	III	1	Yes	No
28	POL_EHV5_III_32	Arabian	III	1	Yes	No
29	POL_EHV5_III_33	Arabian	III	1	Yes	No
30	POL_EHV5_III_34	Arabian	III	1	Yes	No
31	POL_EHV5_III_35	Arabian	III	1	Yes	No
32	POL_EHV5_III_36	Arabian	III	1	Yes	No
33	POL_EHV5_IV_37	Hucul horse	IV	2 - 5	No	No
34	POL_EHV5_IV_38	Hucul horse	IV	1	No	No
35	POL_EHV5_IV_39	Hucul horse	IV	2 - 5	No	No
36	POL_EHV5_IV_40	Hucul horse	IV	1	No	No
37	POL_EHV5_IV_41	Hucul horse	IV	1	No	No
38	POL_EHV5_IV_42	Hucul horse	IV	2 - 5	No	No
39	POL_EHV5_IV_43	Hucul horse	IV	2 - 5	No	No
40	POL_EHV5_V_44	Malopolska horse	V	11 - 15	No	No

41	POL_EHV5_V_45	Malopolska horse	V	1	No	Yes
42	POL_EHV5_V_46	Malopolska horse	V	1	No	Yes
43	POL_EHV5_V_47	Malopolska horse	V	2 - 5	No	No
44	POL_EHV5_V_48	Malopolska horse	V	2 - 5	No	No
45	POL_EHV5_V_49	Malopolska horse	V	1	No	No
46	POL_EHV5_VI_50	Polish Halfbred	VI	11 - 15	No	No
47	POL_EHV5_VI_51	Polish Halfbred	VI	< 1	No	No
48	POL_EHV5_VI_52	Polish Halfbred	VI	16+	No	No
49	POL_EHV5_VII_53	Wielkopolska horse	VII	11 - 15	No	No
50	POL_EHV5_VII_54	Wielkopolska horse	VII	2 - 5	No	No
51	POL_EHV5_VII_55	Wielkopolska horse	VII	11 - 15	No	No
52	POL_EHV5_VII_56	Wielkopolska horse	VII	2 - 5	No	No
53	POL_EHV5_VII_57	Wielkopolska horse	VII	2 - 5	No	Yes
54	POL_EHV5_VII_58	Wielkopolska horse	VII	2 - 5	No	No
55	POL_EHV5_VII_59	Wielkopolska horse	VII	2 - 5	No	No
56	POL_EHV5_VII_60	Wielkopolska horse	VII	< 1	No	No
57	POL_EHV5_VII_61	Wielkopolska horse	VII	< 1	No	No
58	POL_EHV5_VII_62	Wielkopolska horse	VII	< 1	No	No
59	POL_EHV5_VII_64	Wielkopolska horse	VII	2 - 5	No	No
60	POL_EHV5_VIII_66	Wielkopolska horse	VIII	2 - 5	No	No
61	POL_EHV5_VIII_67	Wielkopolska horse	VIII	6 - 10	No	No
62	POL_EHV5_VIII_68	Wielkopolska horse	VIII	< 1	No	No
63	POL_EHV5_VIII_69	Wielkopolska horse	VIII	6 - 10	No	No
64	POL_EHV5_IX_70	Wielkopolska horse	IX	< 1	No	No
65	POL_EHV5_IX_71	Wielkopolska horse	IX	< 1	No	Yes
66	POL_EHV5_X_74	Silesian horse	X	6 - 10	No	No
67	POL_EHV5_X_75	Silesian horse	X	2 - 5	No	No
68	POL_EHV5_X_76	Silesian horse	X	6 - 10	No	No
69	POL_EHV5_XI_77	Thoroughbred	XI	1	No	No
70	POL_EHV5_XI_78	Thoroughbred	XI	1	No	No
71	POL_EHV5_XI_79	Thoroughbred	XI	1	No	No
72	POL_EHV5_XI_80	Thoroughbred	XI	11 - 15	No	No
73	POL_EHV5_XI_81	Thoroughbred	XI	< 1	No	Yes
74	POL_EHV5_XI_82	Thoroughbred	XI	11 - 15	No	Yes
75	POL_EHV5_XI_83	Thoroughbred	XI	11 - 15	No	No
76	POL_EHV5_XI_84	Thoroughbred	XI	6 - 10	No	No
77	POL_EHV5_XI_85	Thoroughbred	XI	11 - 15	No	No
78	POL_EHV5_XI_86	Thoroughbred	XI	1	No	No
79	POL_EHV5_XI_87	Thoroughbred	XI	1	No	No
80	POL_EHV5_XI_88	Thoroughbred	XI	1	No	No
81	POL_EHV5_XII_89	Polish Coldblood	XII	1	No	No
82	POL_EHV5_XII_90	Polish Coldblood	XII	1	No	No
83	POL_EHV5_XII_91	Polish Coldblood	XII	1	No	No
84	POL_EHV5_XII_92	Polish Coldblood	XII	1	No	No
85	POL_EHV5_XII_93	Polish Coldblood	XII	1	No	No

86	POL_EHV5_XII_94	Polish Coldblood	XII	1	No	Yes
87	POL_EHV5_XII_95	Polish Coldblood	XII	1	No	Yes
88	POL_EHV5_XII_96	Polish Coldblood	XII	1	No	Yes
89	POL_EHV5_XIII_97	Polish Konik	XIII	6 - 10	No	No
90	POL_EHV5_XIII_98	Polish Konik	XIII	2 - 5	No	No
91	POL_EHV5_XIII_99	Polish Konik	XIII	1	No	No
92	POL_EHV5_XIII_100	Polish Konik	XIII	2 - 5	No	No
